# Three sets of low-intensity resistance exercises with slow movement and tonic force generation cause more muscular fatigue

**DOI:** 10.1186/s40101-025-00401-x

**Published:** 2025-09-30

**Authors:** Takashi Yamashita, Yulong Ren, Yuta Kosuge, Eisuke Ochi

**Affiliations:** 1 Graduate School of Sports and Health Studies, Hosei University, Aihara Machida, 4342, Tokyo, 194-0298 Japan; 2 Faculty of Bioscience and Applied Chemistry, Hosei University, 3-7-2 Kajino, Koganei, Tokyo, 184-8584 Japan

**Keywords:** Slow training, Muscle fatigue, Electromyography analysis, Root mean square, Mean power frequency

## Abstract

**Purpose:**

There are no previous reports investigating the effects of different set numbers in low-intensity resistance exercise with slow movement and tonic force generation (LST) on muscle fatigue using surface electromyography (sEMG). This study aimed to examine muscle fatigue induced by one set and three sets of LST and to compare the impact of set differences on muscle activity by comparing LST with traditional high-intensity resistance exercise (TRAD).

**Methods:**

Twenty-three healthy male students participated in this study. After 1RM testing in each leg was completed, participants were randomly assigned to either a group performing one set of exercises (*n* = 10) or a group performing three sets (*n* = 13). Each participant performed the LST protocol (50% 1RM) and the TRAD protocol (80% 1RM) with single leg extension until failure. The LST protocol consisted of a 3-s concentric, a 1-s isometric, and a 3-s eccentric phase. In contrast, the TRAD protocol consisted of a 1-s concentric, a 1-s eccentric, and a 1-s rest phase. For the three-set group, a 2-min rest interval was provided between sets. The outcome measures included maximal isometric knee extension torque (MVC) before and after exercise, root mean square (RMS), and mean power frequency (MPF) values recorded during the exercises.

**Results:**

No significant differences in MVC were observed between the type of exercise or the number of sets. Similarly, no significant differences in the RMS during the exercise were observed across exercise types or number of sets. On the other hand, with the significant interaction of MPF (*p* = 0.001, *η*_*p*_^*2*^ = 0.399), there was a significant difference in three sets of LST compared to one set of LST (*p* = 0.012, *d* = 1.16) and three sets of TRAD (*p* < 0.001, *d* = 0.93).

**Conclusion:**

Our findings suggest that performing three sets of LST induces significant muscle fatigue. Therefore, we speculate that performing three sets of LST may lead to the accumulation of metabolic stress and thereby cause muscle fatigue.

## Introduction

The human body sustains vital functions by adapting to various environmental conditions. This adaptive capacity is further enhanced through resistance training (RT) and exercise, which is crucial in improving and maintaining physical function. It is widely recognized that consistent and appropriately applied stimuli to skeletal muscles by RT lead to hypertrophy and enhancements in muscle strength, highlighting its importance in promoting musculoskeletal adaptation. Performing RT at an intensity of approximately 60–80% of one-repetition maximum (1RM) is widely accepted as the optimal range to induce muscle hypertrophy [1] and is commonly taken to training as traditional high-intensity resistance exercise (TRAD). Several studies have demonstrated that this intensity is most effective for promoting increases in muscle size and strength [2–4]. Previous research suggests that the interplay between mechanical stress and cellular responses, such as muscle damage and swelling, plays a crucial role in activating anabolic signaling pathways and the proliferation of muscle and satellite cells, which are integral to the muscle adaptation process [5]. To optimize the training outcomes, however, additional considerations must include training frequency, volume, rest intervals, lifting speed, and sufficient recovery from fatigue.

Meanwhile, previous studies have demonstrated that muscle hypertrophy can also occur with relatively low loads, ranging from 30 to 50% of 1RM, provided that the exercises are performed to failure [6]. For instance, Tanimoto and Ishii [7] showed that hypertrophic adaptations are achievable at intensities as low as 50% 1RM. This method, known as low-intensity resistance training with slow movement and tonic force generation (LST), involves performing resistance exercises with slow movement (approximately 7 s per repetition) while maintaining constant muscle tension throughout the movement. It showed that longer muscle contractions such as LST cause a lower oxygen environment and accumulation of metabolites, thereby resulting in increased hormonal responses [8, 9], reduced muscle oxygen levels [7, 10], and elevated blood lactate levels [11–13]. Moreover, it has been shown that LST is equally effective in increasing muscle mass and strength across both younger and older adults, making it an adaptable approach applicable to diverse populations safely [14–17]. However, most of the previous studies have focused on the chronic effects of LST, while limited data are available regarding the acute muscular responses. Understanding the neuromuscular response after one or more sets of exercises is crucial for identifying how fatigue develops under different training sets.

Muscular fatigue is characterized by a reduced ability to generate maximal force during exercise over time [18]. It can be partly explained by some factors, such as acute change in the neuromuscular system, which arises from metabolic stress, muscular oxygen reduction, metabolite accumulation, and dysfunction in the central and/or peripheral neuromuscular systems [19, 20]. Surface electromyography (sEMG) offers a noninvasive alternative for evaluating skeletal muscle activity during exercise [21–23]. By analyzing the electromyogram waveform, sEMG provides an objective assessment of muscle fatigue during continuous exercise. In particular, the mean power frequency (MPF) obtained from sEMG has been shown to effectively display muscle fatigue by tracking shifts to lower frequency bands during exercise [24]. Previous studies have investigated that MPF significantly decreases during low-intensity resistance training, which was similar to TRAD, speculating fatigue-related contractile dysfunction in fast-twitch muscle fibers [25–27]. In addition to MPF, root mean square (RMS) is another widely used index in sEMG analysis. RMS reflects the amplitude of the EMG signal and is associated with the level of muscle activation, including motor unit recruitment and firing rate. Several studies have reported that RMS increases progressively during continued muscle activity, indicating enhanced motor unit recruitment or compensatory neural drive in response to fatigue [21, 28, 29]. Monitoring both MPF and RMS enables a more comprehensive understanding of neuromuscular fatigue, as they represent different aspects of the fatigue process—MPF and RMS primarily reflecting changes in motor unit behavior. Despite these findings, it remains unclear whether performing one set of either LST or TRAD is sufficient to induce neuromuscular fatigue, or whether multiple sets are required to produce meaningful muscular responses. Clarifying this point is crucial for optimizing training efficiency and understanding the dose–response relationship in resistance exercise.

Therefore, we aimed to examine the effect of one set or three sets on skeletal muscle fatigue during LST, compared to TRAD in this study. We hypothesized that three sets of LST would lead to greater muscle fatigue than one set of LST and TRAD.

## Methods

### Participants

Twenty-three healthy male students (age =23.7 ± 3.6 years, height = 172.0 ± 5.2 cm,weight = 72.1 ± 9.8 kg, %body fat = 19.3 ± 5.4 %,BMI = 24.4 ± 3.1 kg/m^2^) participated in this study. They were all engaged in RT for at least 1 year and had no previous leg or knee injuries. Before the study began, participants were randomly allocated to one-set exercise group or three-set exercise group (one set: *n* = 10, age = 24.6 ± 4.5 years, height = 174.9 ± 6.2 cm, weight = 74.0 ± 10.3 kg, %body fat = 21.2 ± 5.6 %, BMI = 24.1 ± 2.9 kg/m^2^; three sets: *n* = 13, age = 23.1 ± 2.8 years, height = 169.8 ± 3.0 cm, weight = 70.7 ± 9.5 kg, %body fat = 17.9 ± 5.0%, BMI = 24.5 ± 3.3 kg/m^2^). This study was approved by the Ethics Committee of the Graduate School of Sports and Health Sciences, Hosei University (ID: 2023_25).

### Experimental design

The subjects performed single-leg knee extension exercises using a leg extension machine (CYBEX, USA). Participants were randomly assigned to either the one-set or three-set group, making set number a between-subject factor. Each participant performed both exercise protocols (LST and TRAD) on separate legs, making exercise type a within-subject factor. The assignment of protocol to leg (left or right) was randomized. Exercise was performed within the range of 0–90° of knee flexion. In the LST condition, exercises were performed at a 50% 1RM with a total of 7-s repetitions, consisting of 3 s of concentric contraction (lifting), a 1-s isometric contraction, and 3 s of eccentric contraction (lowering), maintaining constant muscle tension throughout all phases. In contrast, the TRAD condition utilized 80% 1RM with a total of 3-s repetitions consisting of 1 s for eccentric contraction, no pause, 1 s for concentric contraction, and 1 s of relaxation. This protocol was followed by methodology from Tanimoto and Ishii [7]. Before each exercise session, participants completed a standardized warm-up consisting of 10 repetitions at 20% 1RM, 5 repetitions at 40% 1RM, and 3 repetitions at 60% 1RM. During the exercise sessions, participants kept their movement tempo using verbal cues from the examiner and a metronome. Both LST and TRAD exercises were provided with verbal instruction and encouragement from the practitioner in each set, and participants performed until they were unable to complete a knee extension. For the three-set group, 2 min of rest periods were provided in between sets. An interval of over 30 min was provided between the two exercise protocols (LST and TRAD), including the time required for repositioning and reattaching the sEMG electrodes.

**Fig. 1:**
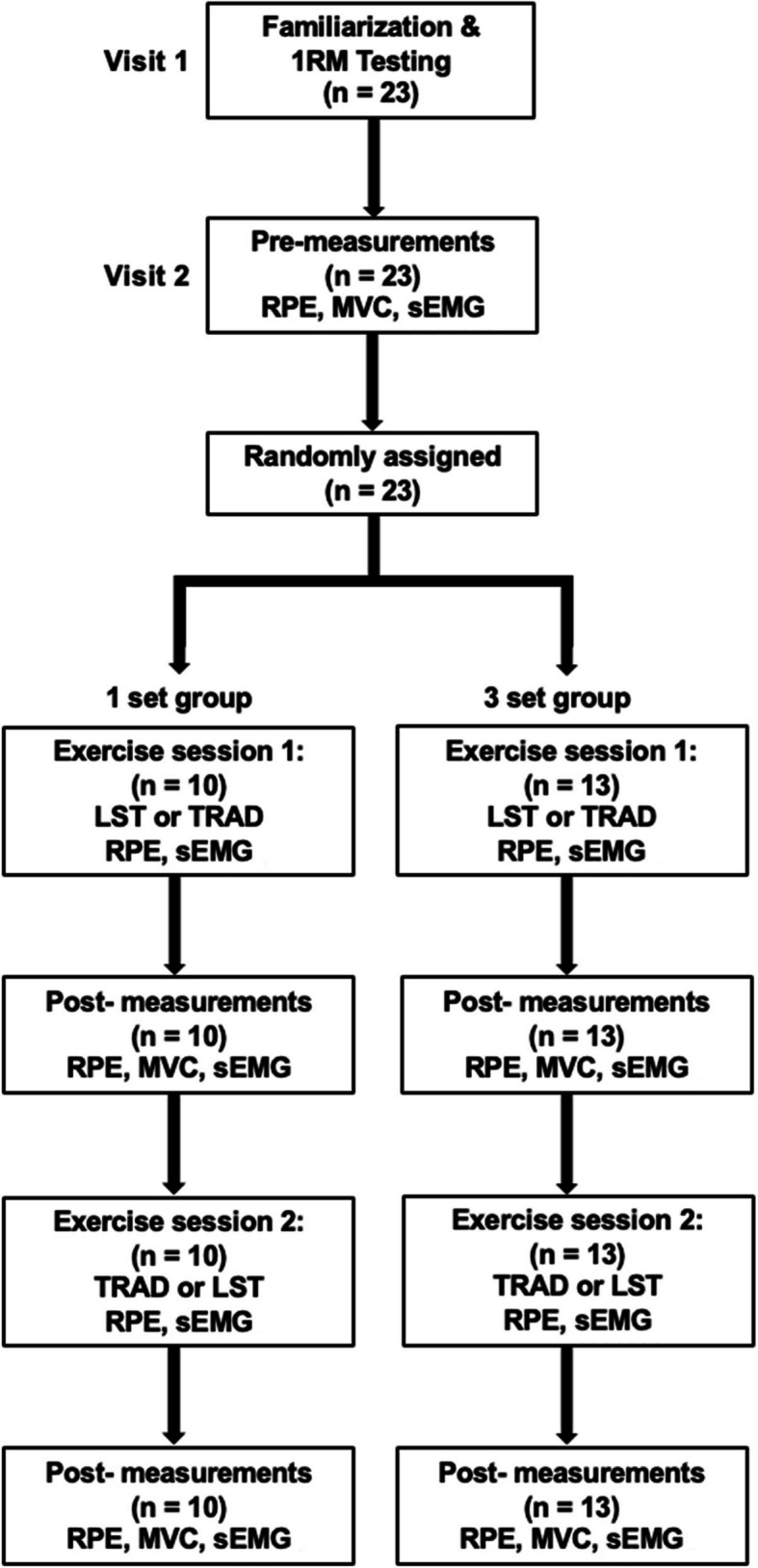
The flow diagram of the experimental protocol. 1RM, one-repetition maximum; RPE, ratings of perceived exertion; MVC, maximal isometric knee extension torque; sEMG, surface electromyography

To evaluate muscle fatigue and exercise outcome, the dependent variables were measured, including maximum voluntary contraction torque (MVC), root mean square (RMS) and MPF from sEMG, and ratings of perceived exertion (RPE). A flow chart of the experimental protocol is described in Fig. 1.

### Maximum voluntary contraction torque (MVC)

MVC during isometric knee extension was assessed before and immediately after each exercise using a custom-made torque dynamometer. Participants were positioned with the knee joints flexed at 90°, and the hip joint flexed at 120° between the trunk and the thigh. Each participant performed three MVC trials, each lasting 3 s, with a 60-s rest period between trials. The peak torque was recorded for each trial, and the average of the three peak torque values was calculated to represent the MVC. The change of MVC from pre- to post-exercise was calculated as ΔMVC. This measurement was based on a previous study [30].

### Surface electromyography (sEMG)

The sEMG was recorded during both MVC testing and exercise using a wireless system (Cometa PICO, COMETA, Italy). To reduce skin resistance, body hair was shaved, and the skin surface was cleaned with alcohol before electrode placement. Wireless bipolar electrodes (Kendall™ H124SG, Cardinal Health, Japan) were positioned on the rectus femoris (RF) muscle from 15 cm above the patella. The raw sEMG signal was sampled at 2 kHz with a band-pass filter set at 10–500 Hz, and the waveform was amplified 1000 times. Data acquisition was performed using a wireless system (WavePlus, COMETA, Italy), and all signal analysis was conducted in biosignal processing software (LabChart 8, ADInstruments, USA).

For signal analysis, the RMS and the MPF values were obtained from the middle 1-s segment of each MVC contraction. Each value from three MVC trials was used to normalize the data from the exercise session. During exercise, the first two and last two repetitions of the concentric phase during both LST and TRAD conditions were analyzed, with the RMS and MPF values for each segment averaged for analysis. The change from the 1 st set to the end of the exercise (1st set of 3rd set) was calculated as ΔRMS and ΔMPF.

### Ratings of perceived exertion (RPE)

The ratings of perceived exertion (RPE) were assessed before and immediately after each set using the Borg scale. The scale ranges from 6, indicating “no exertion at all,” to 20, representing “maximal exertion,” to quantify each participant’s perception of exercise intensity [31].

### Statistical analyses

Statistical analyses were performed using statistical processing software (SPSS version 29.0, IBM, USA). All data are presented as mean ± standard deviation (SD) in the text, tables, and figures. To compare all training variables, ΔMVC, ΔRMS, and ΔMPF of each LST and TRAD condition, a mixed-design two-way ANOVA was used to evaluate the effects of set numbers (between-subjects) and exercise type (within-subjects). If a significant interaction effect was detected, Bonferroni’s post hoc test was performed to detect simple main effects. For effect sizes (ES), partial eta square (*η*_*p*_^*2*^*)* was calculated for interaction and interpretation of small (> 0.01), medium (> 0.06), and large (> 0.14) [32, 33]. In addition, Cohen’s *d* was calculated for post hoc and interpretation of small (> 0.2), medium (> 0.5), and large (> 0.8) [34, 35]. Statistical significance was set at *p* < 0.05.

## Results

### Training session variables

All training independent variables are presented in Table 1. There were significant interactions and differences between groups in total volume (*p* = 0.004, *η*_*p*_^*2*^ = 0.344). Specifically, the post hoc test indicated that three sets of LST were significantly greater than one set of LST (*p* < 0.001, *d* = 1.82) and three sets of TRAD (*p* < 0.001, *d* = 1.41). However, there was no significant interaction in external load, RPE, average repetition, or average time.

**Table 1:** All training variables during LST and TRAD session

		TRAD	LST	Interaction	Set	Exercise	η_p_^2^ value
1RM (kg)	1 set	59.5 ± 10.8	58.1 ± 10.2	0.590	0.884	0.698	0.014
	3 set	59.3 ± 10.9	59.6 ± 14.9				
External load (kg)	1 set	47.4 ± 8.3	29.6 ± 5.0	0.685	0.987	< 0.001	0.008
	3 set	47.0 ± 8.9	29.9 ± 7.2				
RPE	1 set	16.7 ± 1.9	18.1 ± 0.9	0.785	0.287	< 0.001	0.003
	3 set	16.0 ± 1.6	17.6 ± 1.4				
Average repetetion (n)	1 set	8.2 ± 2.0	9.9 ± 2.1	0.168	0.346	< 0.001	0.088
	3 set	6.2 ± 2.3	9.9 ± 4.4				
Average time (sec)	1 set	28.3 ± 7.2	75.0 ± 17.7	0.907	0.137	< 0.001	0.001
	3 set	19.2 ± 4.3	67.0 ± 28.4				
Total volume (n*kg*sec)	1 set	11198.3 ± 4592.5	23467.8 ± 11643.6^†^	0.004	< 0.001	< 0.001	0.335
	3 set	53473.3 ± 36853.3	180075.6 ± 121247.2^#^				

† Significant difference between LST single and triple, p < 0.001.

# Significant difference between LST triple and TRAD triple, p < 0.001.

### Δchange of maximum voluntary contraction torque (MVC)

**Fig. 2:**
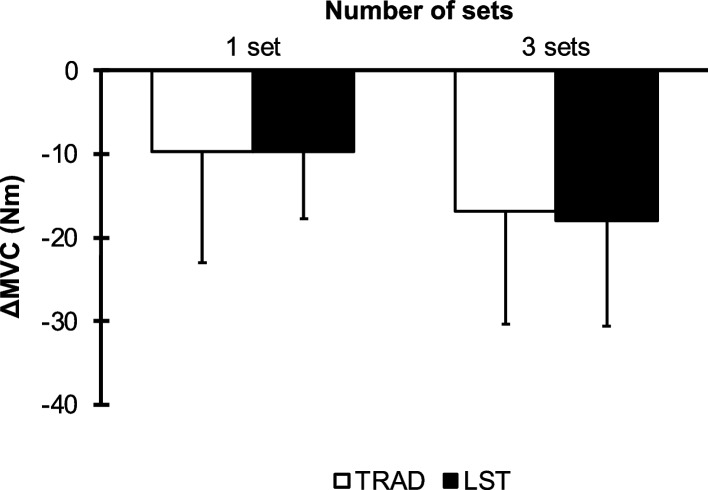
ΔChange in maximum voluntary contraction torque (MVC) from before and after exercise for one set and three sets of TRAD and LST. Data are expressed as mean ± SD

The Δchanges in MVC are presented in Fig. 2. The data showed the decreases in torque in all groups. However, there was no significant difference between groups (*F* [1, 21] = 0.041, *p* = 0.842, *η*_*p*_^*2*^ = 0.002).

### Δchange of root mean square (RMS)

**Fig. 3:**
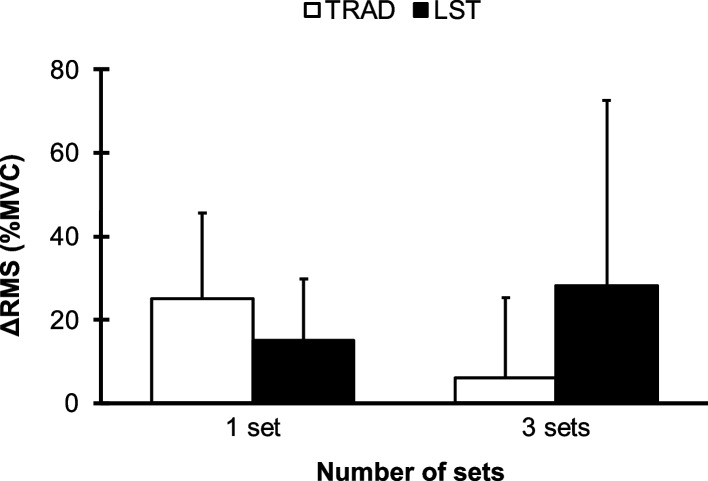
ΔChange in maximum root mean square (RMS) from before and after exercise for one set and three sets of TRAD and LST. Data are expressed as mean ± SD

The Δchanges in RMS are presented in Fig. 3. The data presented the increases in RMS in all groups, while there was no significant difference between groups (*F* [1, 21] = 3.285, *p* = 0.084, *η*_*p*_^*2*^ = 0.135).

### Δchange of mean power frequency (MPF)

**Fig. 4:**
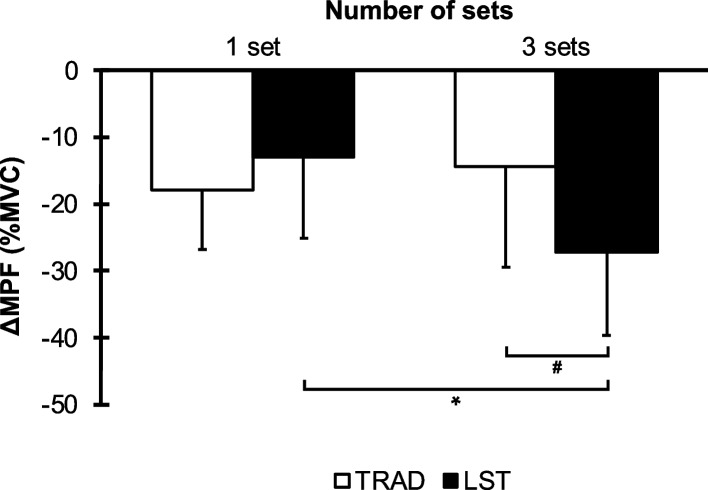
ΔChange in mean power frequency (MPF) from before and after exercise for one set and three sets of TRAD and LST. Data are expressed as mean ± SD. *Significant difference between one set and three sets of LST, *p* < 0.05. #Significant difference between three sets of LST and three sets of TRAD, *p* < 0.05

The Δchanges of MPF are presented in Fig. 4. There was significant interaction between groups (*F* [1, 21] = 13.918, *p* = 0.001, *η*_*p*_^*2*^ = 0.399). The post hoc test showed that three sets of LST were significantly decreased compared to one set of LST (*p* = 0.012, *d* = 1.16) and three sets of TRAD (*p* < 0.001, *d* = 0.93).

## Discussion

In this study, we investigated the difference in muscle fatigue between LST and TRAD by one set or three sets until failure. Regarding training variables, the total volume of three sets for LST was significantly greater than that of one set for LST and three sets for TRAD. In addition, MVC torque and RMS were no significant differences between the groups. However, we found that MPF was significantly decreased in three sets of LST than in one set of LST and three sets of TRAD. Taken together, these findings partially support our hypothesis and suggest that performing multiple sets of LST may contribute to greater muscle fatigue, particularly as indicated by the decrease in MPF.

The present study found that the difference in ΔRMS was not significant regardless of the number of sets or exercise intensity. However, we observed that ΔRMS showed moderate effect size in LST (*η*_*p*_^*2*^ = 0.135). Since previous studies indicate that performing three sets of low-intensity exercise to failure leads to an increase in EMG amplitude comparable to TRAD [25, 36], LST may be a valuable option for producing neuromuscular activation comparable to traditional high-intensity protocols. Previous systematic reviews reported that low-intensity training has a similar effect on muscle hypertrophy with high-intensity training [37, 38]. In addition, several studies have shown that LST positively contributes to muscle hypertrophy [7, 14–16]. Given that LST is a suitable training method for a wide range of individuals, including young untrained, trained, and elderly populations, we propose that incorporating LST into training plans can serve as a viable alternative.

Our results showed a greater decrease of MPF in three sets of LST, compared to one set of LST and three sets of TRAD. This suggests that overall muscle activation remained stable, while frequency-domain changes occurred. Since a reduction in MPF is often associated with decreased motor unit firing rates or reduced recruitment of high-threshold motor units [24, 36, 39], we suggest that central fatigue may have contributed to this pattern. Previous studies have suggested that central fatigue mechanisms can alter neuromuscular output even in the absence of amplitude changes [40]. While our study did not directly measure central fatigue, this possibility should be considered when interpreting the MPF findings.

This study is the first to demonstrate muscle fatigue during LST at 50% 1RM. In previous studies, Tanimoto and Ishii primarily focused on metabolic stress [7]. Studies comparing low- and high-intensity exercises have consistently shown a decrease in MPF in both groups, with a similar decline even as the number of sets increases [36, 41, 42]. Additionally, previous studies reported that the accumulation of metabolic substances during low-intensity exercise with blood flow restriction induces a significant decrease in MPF compared to TRAD [29]. Therefore, a reduction of MPF in three sets of LST has been amplified by prolonged contraction times and additional exercise sets resulting from slower movements, which led to the accumulation of metabolic substances. In addition to the accumulation of metabolites, other mechanisms such as reduced muscle oxygenation and altered neural activation patterns may also contribute to the observed MPF reduction. Although our study did not directly measure these factors, their involvement cannot be excluded and warrants further investigation in future studies.

This study has several limitations. First, the data was obtained from participants who were not experienced with LST. Although participants had an average of 3.8 ± 2.9 years of general training experience and 2.3 ± 1.2 times per week of training frequency, the unfamiliarity with LST was limited to consistently maintaining muscle contractions. Second, this study did not measure the blood pressure and blood lactate levels. Previous research has shown that blood pressure and lactate levels decrease during and after LST, making them valuable metrics for further investigation. Further studies should examine the relationship between acute changes in muscle activity and blood pressure and/or lactate levels to understand the physiological mechanisms of LST. Third, although all participants were thoroughly instructed and encouraged to exert maximal effort during each session, the variation in total training volume was relatively large. This variability may reflect differences in participants’ backgrounds, such as training experience or motivational factors, and should be considered when interpreting the results. These points would provide a more comprehensive understanding of LST’s applicability and effectiveness in varied training contexts.

## Conclusion

In conclusion, we found that three sets of LST caused a decrease in MPF. This result suggests that performing three sets of LST may lead to muscle fatigue. Further research is necessary to investigate the physiological mechanisms underlying muscle fatigue induced by LST, with a particular focus on the relationship between metabolic stress and changes in MPF. The evaluation of central fatigue will enhance our understanding of the whole fatigue processes associated with LST.

## Data Availability

The datasets during and/or analyzed during the current study are available from the corresponding author upon reasonable request.
